# Experimental Laboratory Study on the Acoustic Response Characteristics of Fluid Flow in Horizontal Wells Based on Distributed Fiber Optic Sensing

**DOI:** 10.3390/s26072248

**Published:** 2026-04-05

**Authors:** Geyitian Feng, Zhengting Yan, Jixin Li, Yang Ni, Manjiang Li, Zhanzhu Li, Xin Huang, Junchao Li, Qinzhuo Liao, Xu Liu

**Affiliations:** 1 National Key Laboratory of Petroleum Resources and Engineering, China University of Petroleum-Beijing, Beijing 102249, China; 2025315105@student.cup.edu.cn (G.F.); 2024310148@student.cup.edu.cn (Z.Y.); 2024215153@student.cup.edu.cn (Y.N.); 2 College of Mechanical Engineering, Xi’an Shiyou University, Xi’an 710065, China; ljx_humble@163.com (J.L.); lijunchao@xsyu.edu.cn (J.L.); 3 CNPC Chuanqing Drilling Engineering Company Limited, Xi’an 710065, China; zjssbmj@cnpc.com.cn (M.L.); zqjdlzz@cnpc.com.cn (Z.L.); 4 SINOPEC Petroleum Exploration and Production Research Institute, Beijing 102206, China; huangxin2020.syky@sinopec.com; 5 College of Petroleum Engineering & Geosciences, King Fahd University of Petroleum and Minerals, Dhahran 31261, Saudi Arabia; liu.xu@kfupm.edu.sa

**Keywords:** DAS, acoustic response characteristics, laboratory experiments, single-phase flow, characteristic frequency bands, FBE, regression analysis

## Abstract

Distributed acoustic sensing (DAS) has been widely applied to injection–production profile monitoring in horizontal wells because it provides continuous full-wellbore coverage, real-time acquisition, and straightforward long-term deployment. In practical downhole operations, however, DAS measurements are frequently compromised by optical-signal attenuation, loss of fiber–casing/formation coupling, and environmental noise. Meanwhile, the mechanisms governing flow-induced acoustic responses remain insufficiently understood, which continues to impede quantitative diagnosis and interpretation of injection–production profiles based on DAS data. To address these challenges, this study performed controlled laboratory-scale physical simulation experiments of single-phase flow in a horizontal wellbore, systematically investigating DAS acoustic responses under two wellbore diameters (25 mm and 50 mm) and a range of flow velocities. Power spectral density (PSD) was derived using the fast Fourier transform to identify flow-sensitive characteristic frequency bands, and frequency-band energy (FBE) was further used to establish an optimal quantitative relationship with flow velocity. The results show that: (1) DAS energy is dominated by low-frequency components (<100 Hz), with the total energy increasing nonlinearly as flow velocity rises, accompanied by a progressive broadening of the characteristic bands; (2) the feature bands identified using an adaptive method based on energy difference statistics applied to PSD frequency-domain features exhibit a higher signal-to-noise ratio and greater physical clarity than traditional wide frequency bands; furthermore, by employing a feature band merging strategy, the distribution characteristics of flow energy can be captured more comprehensively; and (3) FBE exhibits a strong nonlinear dependence on flow velocity, with a power-law model delivering the best theoretical fit, whereas a cubic model (FBE ∝ *V*^3^) achieves high accuracy and robustness for practical applications. The proposed workflow—“PSD peak identification–characteristic band delineation–FBE regression”—establishes a methodological foundation for quantitative DAS-based monitoring of horizontal-well injection–production profiles in both laboratory and field settings, and it provides a basis for subsequent intelligent monitoring and interpretation under multiphase-flow conditions.

## 1. Introduction

Distributed fiber optic sensing employs optical fibers as a continuous sensing medium, enabling high-precision, real-time, and spatially continuous measurements of temperature, strain, and vibration along extended wellbore intervals [1,2]. Compared with conventional downhole surveillance approaches that rely on point sensors, current meters, or conveyance-based logging tools, DOFS offers clear advantages by avoiding sparse spatial sampling, high operational and re-entry costs, sensitivity to well conditions, and the increased interpretation uncertainty commonly encountered in complex horizontal wells. Conventional current meters or flow meters generally provide accurate local measurements under controlled conditions; however, their applicability in unconventional wells is often constrained by limited spatial coverage, deployment complexity, and difficulty in capturing highly heterogeneous or multipoint inflow behavior. These limitations become more pronounced in unconventional wells featuring long laterals, strong heterogeneity, or multiple inflow points, where conventional production logging faces elevated uncertainty in both execution and flow-allocation diagnostics [3,4]. Owing to its immunity to electromagnetic interference, tolerance to high-temperature/high-pressure environments, suitability for long-term downhole deployment, and continuous full-wellbore coverage [5], distributed fiber-optic monitoring has evolved from an auxiliary technique into an essential surveillance tool [6,7]. In this sense, DOFS should be regarded not as a direct replacement for conventional current meters, but as a complementary technology that is particularly advantageous for distributed flow characterization and real-time wellbore surveillance.

Within the distributed fiber-optic sensing family, DAS is typically based on Rayleigh backscattering and phase-sensitive optical time-domain reflectometry (ϕ-OTDR) [8]. In this framework, minute strain perturbations along the fiber are converted into interpretable vibration/acoustic signals, enabling real-time acquisition of distributed strain and vibration responses along the wellbore [9]. In recent years, DAS has been extensively applied to injection–production profile monitoring, post-fracturing performance evaluation, and wellbore integrity assessment [10,11]. In practical field development, however, DAS measurements remain vulnerable to the combined effects of signal attenuation and demodulation-chain noise, variations in fiber–casing/formation coupling conditions, equipment-induced vibrations from pumps and valves, and ambient surface noise [12]. Meanwhile, the generation and transmission of flow-induced acoustic radiation within a wellbore typically involve tightly coupled multi-physics processes—fluid dynamics, tubular structural vibration, and wellbore boundary conditions—so that the mapping between “signal features” and “flow parameters” becomes strongly nonlinear and highly dependent on operating conditions [13]. Consequently, controlled laboratory experiments for wellbore injection–production profile monitoring are necessary to establish repeatable datasets and a quantitative interpretation framework for flow-induced acoustic responses while minimizing confounding field disturbances [14,15,16].

To systematically investigate DAS acoustic responses under wellbore flow conditions, various sophisticated laboratory-scale physical simulation facilities have been developed [17]. Martinez et al. (2014) [18] provided an early experimental verification by constructing a vertical wellbore setup to evaluate the feasibility of DAS-based monitoring in hydraulically fractured producing wells. Finfer et al. (2014) [19] utilized a closed-loop circulation system to perform gas–liquid two-phase experiments, enabling the analysis of vortex structures and sound-speed-related characteristics. Similarly, Liu (2019) [20] constructed a dedicated horizontal-well physical simulation facility to capture two-phase flow acoustic signatures. To simulate more complex downhole conditions, Chen et al. (2015) [21] developed a production simulation apparatus specifically for hydraulically fractured wells, investigating the acoustic signatures generated by water–sand two-phase flow through perforation structures. More recently, Zen et al. (2025) [22] developed a full-scale DAS wellbore-flow monitoring facility, successfully conducting single-phase gas and gas–water two-phase experiments to validate production profile monitoring.

Alongside the advancement of physical simulation facilities, substantial progress has been made in signal processing and quantitative interpretation methodologies to map DAS acoustic features to flow parameters. in’t Panhuis et al. (2023) [23] conducted spectral analysis via Fourier transformation and extracted audio features using a filter-bank energy approach to establish a flow-noise correlation model. Building on this framework, Li (2022) [24] combined filter-based denoising with Fourier-feature extraction to propose a quantitative relationship between fracture flow rate and sound pressure level. Luo et al. (2026) [25] further extracted effective frequency–amplitude features via fast Fourier transform and digital filtering to model DAS acoustic energy against wellbore flow velocity under gas–water conditions. In pursuit of higher signal-to-noise ratios, Li (2020) [26] applied moving-average smoothing and wavelet-packet denoising alongside Fourier transformation to develop a mathematical model predicting fracture inflow and outflow. With the increasing complexity of DAS data, machine learning techniques have been integrated into the processing workflow. Liu et al. (2024) [27] combined Fourier and time–frequency feature extraction with machine learning to build a water-cut interval classification model. Similarly, Fang et al. (2025) [28] achieved intelligent flow-regime identification by integrating time-domain, frequency-domain, and time–frequency features with machine learning algorithms. Furthermore, Liu (2020) [29] optimized the signal-processing workflow by introducing an artificial neural network (ANN) to establish an intelligent recognition model for two-phase flow states

However, there are currently issues with the quantitative interpretation method of flow-induced acoustics in horizontal wellbores based on DAS. Key fundamental issues—such as power spectral density characteristics, the delineation of characteristic frequency bands, and the quantitative relationship between frequency band energy and flow velocity—require systematic preliminary research under controlled single-phase conditions. Furthermore, existing literature primarily focuses on the acoustic response of perforation outflow, while research on the acoustic characteristics of internal pipeline flow (particularly flow within horizontal wellbores) remains relatively limited. The flow field structures and excitation mechanisms of these two scenarios differ fundamentally. This study starts with single-phase flow and aims to construct a systematic methodological framework from signal processing to quantitative modeling. The research results can provide experimentally verifiable evidence and methodological support for the quantitative interpretation of horizontal well injection-production profile monitoring using DAS.

## 2. Experimental Design

### 2.1. Experimental Apparatus

**Figure 1:**
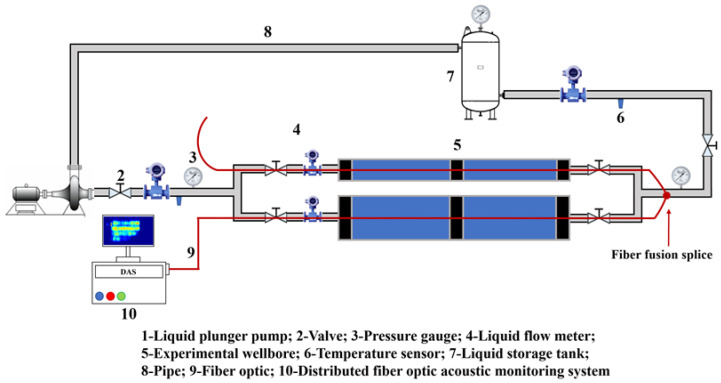
Laboratory experimental setup for distributed fiber-optic monitoring of injection–production profiles in a horizontal wellbore.

This study was carried out on a laboratory-scale distributed fiber-optic monitoring platform for horizontal-well injection–production profiling, where single-phase water flow-induced noise responses were measured using DAS. As shown in Figure 1, the experimental setup consists of three main components: a DAS monitoring module, a simulated wellbore module, and a fluid injection–return module.

The DAS monitoring module comprises an intelligent distributed acoustic sensing system and a single-mode optical fiber for real-time acquisition and recording of acoustic signals along the wellbore. A commercial coherent optical time-domain reflectometry (C-OTDR)-based DAS interrogator (MS-DAS, Wuhan Megasense Technology Co., Ltd., Wuhan, China) was used in continuous acquisition mode. The interrogator operates at a typical center wavelength of 1550 nm with an optical output power of approximately 10 mW. It was connected to a standard single-mode optical fiber with a refractive index of 1.468. To capture flow-induced noise effectively, the optical pulse width was set to 80 ns. The system recorded acoustic data with a spatial resolution of 1.02 m (with the gauge length equal to the spatial resolution) and a temporal sampling frequency of 2 kHz. In addition, anti-aliasing low-pass filtering was enabled in the DAS acquisition chain before digitization to suppress frequency components above the Nyquist limit and prevent out-of-band mechanical noise from folding into the analyzed spectrum.

The simulated wellbore module includes two transparent poly (vinyl chloride) (PVC) pipe sections, each 8 m long, with inner diameters of 25 mm and 50 mm, respectively, representing different wellbore-size conditions. These laboratory diameters were selected to provide scaled and controlled flow conditions rather than a strict one-to-one reproduction of field tubular geometry. From a dimensional-analysis perspective, the flow-induced DAS response is more fundamentally governed by dimensionless parameters, such as the Reynolds and Strouhal numbers, than by absolute diameter alone. Thus, the two diameter cases provide a practical basis for examining diameter-dependent acoustic behavior while preserving the essential flow physics.

The fluid injection–return module is equipped with a plunger pump, a pulsation dampener (pressure-stabilization tank), a high-pressure manifold, and precision temperature and pressure sensors to maintain a stable flow field. To reduce interference from external mechanical vibrations, the plunger pump was isolated using a high-density foam pad beneath its base. The optical fiber was continuously deployed along the wellbore axis, and the two pipe sections were connected in series within the same optical path by fusion splicing. This configuration allowed synchronous acquisition of DAS responses under both diameter conditions using identical interrogator settings, providing a consistent basis for comparative analysis.

### 2.2. Experimental Program

To elucidate the DAS acoustic response characteristics of single-phase flow in a horizontal wellbore under different diameters (25 mm and 50 mm) and flow-rate conditions (1.06–2.58 m^3^/h), a unidirectional-flow experiment was conducted using the laboratory wellbore flow facility. A plunger pump served as the driving source, and the injection flow rate was regulated by adjusting the pump rotational speed (1100–2400 rpm) in combination with a flowmeter. For each pipe diameter, the flow velocity was increased stepwise from low to high through a series of discrete operating conditions (Table 1). After each pump-speed adjustment, a stabilization period was allowed to ensure steady flow conditions. Once the flowmeter reading became stable, distributed acoustic data and hydraulic parameters were synchronously acquired and stored. This design enables systematic evaluation of the effects of diameter and flow-rate variations on DAS acoustic-energy distribution, spectral characteristics, and along-well response differences under consistent experimental conditions. The resulting dataset provides the basis for subsequent analysis of acoustic response patterns and mechanistic interpretation.

To maintain experimental consistency throughout the test campaign, tap water was used as the working fluid in all experiments. The tests were conducted on the same platform, using the same water source and a consistent data-acquisition procedure after the flow had stabilized. Under winter laboratory conditions, the water temperature was approximately 10–20 °C. Because water properties and acoustic propagation characteristics may vary with temperature, the possible magnitude of this effect was further evaluated in terms of sound-speed variation. At a pressure of 1.1 MPa, the sound speed in water is approximately 1448.9 m/s at 10 °C and 1484.0 m/s at 20 °C, corresponding to a difference of 35.1 m/s, or about 2.37%. This variation is considered to be within a controllable range for the comparative laboratory analysis conducted in this study. The influence of bubbles was also considered. Since the simulated wellbore section was transparent, data acquisition was initiated only after no visible bubbles remained in the pipe, thereby reducing the potential effects of entrained air on acoustic propagation.

## 3. Methods

This section presents a complete data-processing workflow for the DAS measurements acquired in the wellbore-flow experiments, covering raw-signal preprocessing, the PSD analysis, characteristic frequency-band delineation, FBE extraction, and the development of velocity–response models. Specifically, PSD is estimated via the fast Fourier transform (FFT) to identify spectral-peak features and to quantify how these features evolve with flow velocity. Based on the peak behavior, flow-sensitive frequency bands are defined, within which FBE is computed and subsequently regressed against flow velocity to establish a quantitative relationship model.

### 3.1. PSD Estimation Based on FFT

The DAS measurements are discrete-time signals *x*(*n*) recorded in the time–distance domain. Before digitization, anti-aliasing low-pass filtering was applied in the DAS acquisition chain to attenuate frequency components above the Nyquist frequency. This step helps prevent high-frequency mechanical vibrations from folding into the low-frequency band of interest. Prior to spectral analysis, each time-domain frame was multiplied by a Hamming window to reduce spectral leakage caused by finite-length truncation. In our preliminary experiments, we compared the effects of rectangular, Hanning, and Hamming windows on power spectral density estimation. The rectangular window introduces significant high-frequency leakage, leading to distortion of the DAS signal. Both the Hanning and Hamming windows exhibit excellent sidelobe suppression performance. The former has better overall sidelobe attenuation, while the latter has stronger suppression of the first sidelobe, resulting in better isolation of strong single-frequency components. Taking into account the spectral characteristics of the DAS signal, we chose the Hamming window for Fourier transformation in this paper [30,31], Hamming window [32]:

(1) $$w(n)=0.54-0.46\cos(\frac{2\pi n}{N-1})$$

where *n* is the time index and *N* is the frame length. The windowed signal was then transformed into the frequency domain using FFT [33], a standard and computationally efficient method for spectral decomposition [34]. Fast Fourier transform:

(2) $$X_{w}(k)=\sum_{n=0}^{N-1}x(n)w(n)e^{-j\frac{2\pi nk}{N}}$$

To characterize how acoustic energy is distributed across frequencies and to improve the comparability of different operating conditions, PSD was used as the main frequency-domain descriptor [35]. Compared with the direct amplitude spectrum, PSD better reflects the distribution of signal power over frequency and is therefore more suitable for identifying dominant flow-induced frequency bands and tracking their evolution with flow velocity. On this basis, the characteristic frequency bands were determined from the PSD peak distribution [36].

Power spectral density [37]:

(3) $$S_{xx}(f_{k})=\frac{1}{f_{s}\sum_{n=0}^{N-1}w^{2}[n]}{\left|X_{w}[k]\right|}^{2}$$

where *f_s_* is the sample frequency. The PSD unit is rad^2^/Hz.

### 3.2. Adaptive Feature Band Partitioning Based on Energy Difference Statistics

The physical mechanisms underlying flow-induced acoustic responses in horizontal wellbores are highly complex, involving turbulence, vibrations of the wellbore wall structure, and fluid–structure interaction. At present, it is difficult to theoretically determine a unified set of characteristic frequency bands in advance. Therefore, our selection of characteristic frequency bands is directly guided by the data, rather than imposing a fixed bandwidth in advance, to ensure that the selected bands are physically relevant to the flow-induced excitation under the given experimental configuration. The specific method is as follows:

Compute the first-order difference ΔE of the preprocessed PSD to quantify the local rate of change in energy with frequency; Calculate the mean *μ* and standard deviation *σ* of the absolute values of all differences, and construct an adaptive threshold


(4) τ=α·μ+β·σ

In this study, *α* is the baseline weighting coefficient, which determines the extent to which the mean *μ* contributes to the threshold. When *α* is large, the threshold is primarily controlled by the overall level of fluctuation, resulting in fewer detected feature bands (only the most pronounced changes are retained). *β* is an adjustable sensitivity parameter. By adjusting *β*, the level of detection sensitivity can be controlled: the smaller *β* is, the finer the detected changes; the larger *β* is, the more only the most significant changes are retained. Continuous frequency intervals satisfying |ΔE| > *τ* are marked as feature bands.

### 3.3. FBE Estimation

After obtaining the PSD, FBE was introduced to quantify the energy contribution of flow-induced acoustic responses within a specified frequency interval. In essence, FBE is the integral of PSD over a target band, and its discrete form is obtained by summing PSD values over the corresponding frequency bins. Compared with single-frequency peak values, this band-integrated measure is less sensitive to local spectral fluctuations and better captures the overall strength of flow-related acoustic excitation.

To further reduce the influence of absolute signal-level variation and improve cross-condition comparability, a normalized band-energy ratio was also defined, representing the fraction of total spectral energy contributed by the target band. This normalized expression is conceptually consistent with the power-spectrum sub-band energy ratio and is useful for comparing flow-sensitive bands under different signal amplitudes, coupling conditions, or background noise levels.

For a target band [*f*_1_, *f*_2_], the band energy *E*_[*f*1, *f*2]_ is defined as the integral of the PSD over the band. The unit of FBE is rad^2^ [38]:

(5) $$E_{\left[f_{1},f_{2}\right]}=\int_{f_{1}}^{f_{2}}S_{xx}(f)df$$

For the discrete PSD estimate *S_xx_*(*f_k_*) evaluated on a frequency grid *f_k_* = *k*Δ*f* with frequency resolution Δ*f* = *f_s_*/*N*, Equation (4) can be approximated by a summation [38]:

(6) $$E_{\left[f_{1},f_{2}\right]}\approx \sum_{k=k_{1}}^{k_{2}}S_{xx}(f_{k})\Delta f,\;k_{1}=\frac{f_{1}}{\Delta f},k_{2}=\frac{f_{2}}{\Delta f}$$

To facilitate comparisons across different signal segments or operating conditions, a normalized band-energy ratio can be further defined as [38]:

(7) $$\eta_{\left[f_{1},f_{2}\right]}=\frac{E_{\left[f_{1},f_{2}\right]}}{\int_{0}^{f_{s}/2}S_{xx}(f)df}\approx \frac{\sum_{k=k_{1}}^{k_{2}}S_{xx}(f_{k})\Delta f}{\sum_{k=0}^{N/2}S_{xx}(f_{k})\Delta f}$$

where *η*_[*f*1, *f*2]_ represents the fraction of total spectral energy (within 0 to *f_s_*) contributed by the target band.

In addition, current research also employs methods such as sound pressure level, time-frequency characteristics, and wavelet energy. We conducted a comparison of these methods, as shown in Table 2.

### 3.4. Fitting Method for the FBE–Velocity Relationship

To quantify the relationship between the energy response of distributed fiber-optic acoustic signals within different characteristic frequency bands and the flow velocity in a horizontal wellbore, this study uses FBE as the response variable and the mean flow velocity *V* as the independent variable. Empirical FBE–*V* models are established separately for each selected characteristic band (e.g., 16–25 Hz, 25–38 Hz, and 16–38 Hz). Because the experimental data may exhibit distinct trends—ranging from linear behavior to nonlinear power—law or exponential-type growth—multiple candidate models are evaluated and compared under a unified formulation consistent with the equations adopted in this work, as summarized below.

To capture the monotonic, nonlinear intensification of FBE with increasing velocity, single-term power functions with polynomial orders from one to six are considered [39]:

(8) Linear: FBE=aV

(9) $$\mathrm{Quadratic}:\;\mathrm{FBE}=aV^{2}$$

(10) $$\mathrm{Cubic}:\;\mathrm{FBE}=aV^{3}$$

(11) $$\mathrm{Quartic}:\;\mathrm{FBE}=aV^{4}$$

(12) $$\mathrm{Quintic}:\;\mathrm{FBE}=aV^{5}$$

(13) $$\mathrm{Sextic}:\;\mathrm{FBE}=aV^{6}$$

where *a* is a fitting coefficient.

When the FBE response to flow velocity exhibits a “scaled amplification” pattern, a general power-law model is adopted [40]:

(14) $$\mathrm{FBE}=aV^{b}$$

where *a* and *b* are fitting parameters, and b characterizes the degree of nonlinearity in the response.

When FBE varies with *V* in an exponential manner, an exponential model is used [40]:

(15) $$\mathrm{FBE}=ae^{bV}$$

where *a* and *b* are fitting parameters, and b controls the growth (*b* > 0) or decay (*b* < 0) rate.

Model parameters are estimated using the least-squares criterion. Model parameters were estimated using the least-squares criterion, which minimizes the residual sum of squares between the measured FBE values and the model predictions.

Given 𝑛 experimental data pairs ${\left\{(V_{i},{\mathrm{FBE}}_{i})\right\}}_{i=1}^{n}$, the fitted value is ${\hat{\mathrm{FBE}}}_{i}=f(V_{i};\theta )$, where *θ* denotes the parameter set. The objective function is defined as [41]:

(16) $$\underset{\theta }{\min}\sum_{i=1}^{n}{\left({\mathrm{FBE}}_{i}-{\hat{\mathrm{FBE}}}_{i}\right)}^{2}$$

To evaluate and compare the explanatory power of candidate models, the coefficient of determination *R*^2^ is employed as the goodness-of-fit metric. *R*^2^ represents the proportion of variance in the observations explained by the model; a larger *R*^2^ indicates a better representation of the FBE–V relationship within the considered frequency band. *R*^2^ is defined based on the residual sum of squares (SSE) and the total sum of squares (SST) [41]:

(17) $$\mathrm{SSE}=\sum_{i=1}^{n}{\left({\mathrm{FBE}}_{i}-{\hat{\mathrm{FBE}}}_{i}\right)}^{2}$$

(18) $$\mathrm{SST}=\sum_{i=1}^{n}{\left({\mathrm{FBE}}_{i}-\bar{\mathrm{FBE}}\right)}^{2}$$

where $\bar{\mathrm{FBE}}=\frac{1}{n}\sum_{i=1}^{n}{\mathrm{FBE}}_{i}$ is the sample mean. The coefficient of determination is then

(19) $$R^{2}=1-\frac{\mathrm{SSE}}{\mathrm{SST}}$$

## 4. Results

### 4.1. Background Noise Calibration and Pump-Vibration Assessment

**Figure 2:**
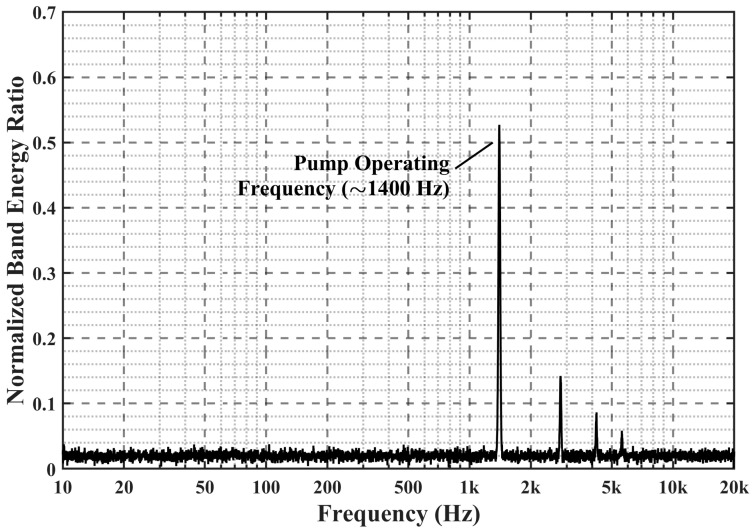
PSD spectrum of the plunger-pump background-noise test.

Furthermore, a rigorous background-noise calibration was conducted prior to the formal flow experiments to isolate the mechanical noise generated by the plunger pump. During this pre-test, the pump was operated while the flow loops were bypassed, and the DAS sampling rate was temporarily increased to 20 kHz (yielding a Nyquist frequency of 10 kHz) to capture a broader acoustic spectrum. As illustrated in Figure 2, the power spectral density analysis revealed that the pump-induced mechanical vibration is mainly concentrated around 1400 Hz, which is well separated from the low-frequency flow-induced response bands analyzed in this study.

For the formal flow experiments, the DAS system was configured with a sampling rate of 2000 Hz to balance data storage and sensitivity to low-frequency flow-induced acoustic signals, corresponding to a Nyquist frequency of 1000 Hz. To avoid aliasing of out-of-band components, anti-aliasing low-pass filtering was applied in the DAS acquisition chain before digitization. Therefore, the pump-related vibration component near 1400 Hz was sufficiently attenuated and did not fold back into the recorded spectrum. In addition, the flow-sensitive characteristic bands identified in the subsequent analysis are mainly concentrated below 100 Hz, far from the pump’s intrinsic vibration frequency. Accordingly, the influence of pump-induced mechanical vibration on the subsequent PSD and FBE analysis can be regarded as negligible.

The measured DAS response may also be influenced by the thermophysical state of the fluid. In this study, tap water was used under an approximate temperature range of 10–20 °C. Temperature variations may slightly affect water properties and, consequently, acoustic propagation characteristics. Residual microbubbles, if present, may also introduce additional attenuation and scattering effects. To minimize these influences, all experiments were performed under similar seasonal and daily time conditions, and data acquisition was initiated only after the flowmeter readings had stabilized and no visible bubbles remained in the transparent pipe section. Under these controlled conditions, the effects of temperature fluctuation and residual microbubbles are considered secondary and do not alter the main comparative conclusions regarding the spectral evolution of DAS signals and the variation trend of FBE with flow velocity.

### 4.2. Effect of Flow Velocity on Signal Frequency

**Figure 3:**
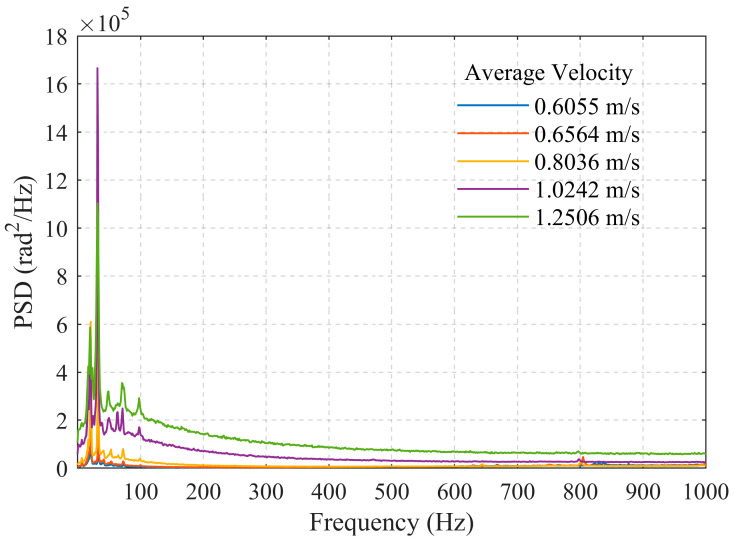
Comparison of PSD at different flow velocities for the 25 mm wellbore.

Figure 3 presents the acoustic frequency-domain energy distribution data for the 25 mm wellbore diameter under different flow velocities (0.6055 m/s, 0.6564 m/s, 0.8036 m/s, 1.0242 m/s, 1.2506 m/s). In the PSD curves, flow-related acoustic signals typically manifest as several prominent peaks. As illustrated, the overall trend shows a clear increase in acoustic energy with rising flow velocity. Within the lower velocity range from 0.6055 m/s to 0.8036 m/s, the growth in energy value was relatively gradual. However, when the velocity further increased to 1.0242 m/s and 1.2506 m/s, the energy values exhibited a marked surge. This indicates a positive correlation between acoustic intensity and flow velocity, aligning with the fundamental physical principle that flow-induced noise energy increases with flow velocity.

Regarding frequency-domain distribution characteristics, the frequency bands of energy concentration under different flow velocities showed distinct differences. At velocities of 0.6055 m/s and 0.6564 m/s, energy was primarily concentrated in the low-frequency region below 50 Hz, with a relatively narrow bandwidth. The energy distribution curve was smooth, exhibiting a dominant dual-peak feature with peaks located near 20 Hz and 32 Hz. As the flow velocity gradually increased to 0.8036 m/s and beyond, while maintaining low-frequency dominance, the energy began to extend into the 50–100 Hz frequency band. Within the 100 Hz range, besides the two main peaks, multiple local minor peaks appeared, indicating enhanced flow disturbances. Concurrently, the energy corresponding to the dual-peak frequencies increased significantly, more than doubling in amplitude compared to lower velocities. The low-frequency band (<100 Hz) consistently dominated the energy distribution. Further analysis of the energy attenuation characteristics with frequency revealed that under low-flow conditions, energy decayed rapidly as frequency increased. In contrast, under high-flow conditions, energy remained at a relatively high level across a broader frequency band (20–100 Hz), with the acoustic energy distribution becoming more continuous and widespread. Subsequently, the energy decay approximated an exponential trend.

**Figure 4:**
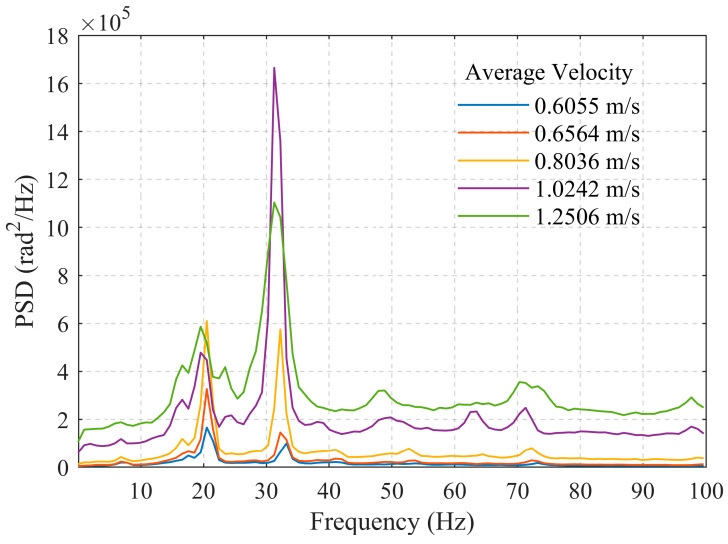
PSD within 0–100 Hz for the 25 mm wellbore.

Figure 4 shows a locally magnified view of PSD within the 0–100 Hz range. It can be observed that as the flow velocity increases, the bandwidth of the dominant peak widens significantly, exhibiting a broadband characteristic with multiple dominant frequencies coexisting. This indicates that the increased flow velocity enhances the turbulent state of the fluid flow, leading to more complex flow structures. Acoustic events such as vortex shedding and fluid-wall collisions become notably more frequent, thereby exciting a richer spectrum of frequency components.

**Figure 5:**
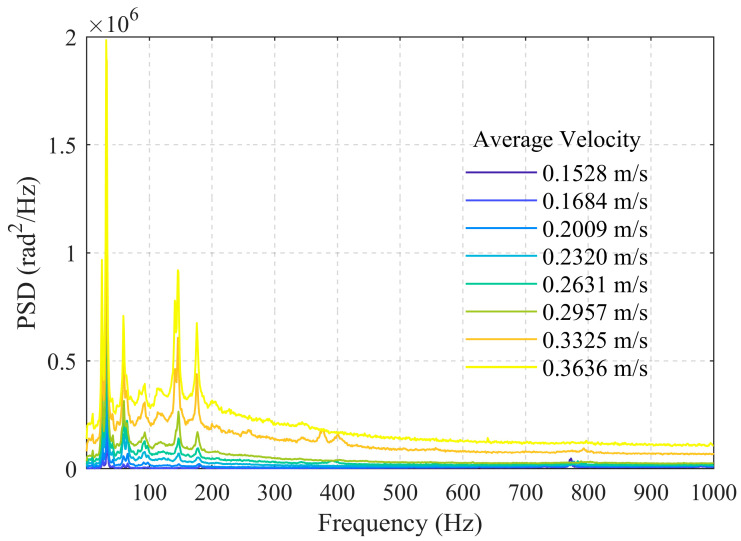
PSD at different flow velocities for the 50 mm wellbore.

**Figure 6:**
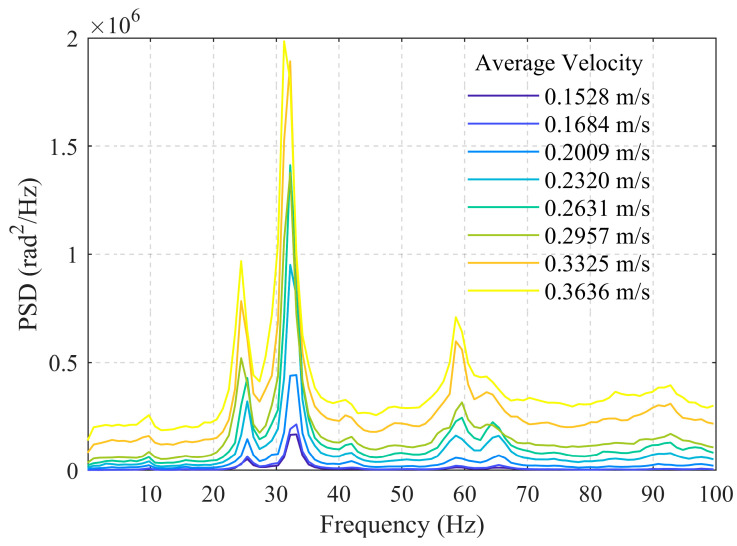
PSD within 0–100 Hz for the 50 mm wellbore.

Figure 5 presents the acoustic frequency-domain energy distribution data for the 50 mm wellbore diameter under different flow velocities (0.1528 m/s, 0.1684 m/s, 0.2009 m/s, 0.2320 m/s, 0.2631 m/s, 0.2957 m/s, 0.3325 m/s, 0.3636 m/s). The results exhibit a phenomenon similar to that observed for the 25 mm wellbore: as the flow velocity gradually increases, the acoustic energy shows a clear incremental rise, with energy concentrated in the low-frequency region. While maintaining low-frequency dominance, the energy begins to extend into the 100–200 Hz frequency band. The difference lies in the low-frequency portion, where three distinct peak frequencies appear, located near 25 Hz, 31 Hz, and 58 Hz. Furthermore, these peak frequencies remain almost unchanged with increasing flow velocity. We hypothesize that this may be due to the experimental system (including the wellbore, etc.) possessing inherent mechanical vibration modal frequencies. When the frequency components of the flow-induced excitation (such as turbulent pressure fluctuations) cover or approach these natural frequencies, structural resonance can occur. This leads to a significant amplification of the vibrational response (amplitude) at these frequencies, manifesting as prominent, sharp peaks in the spectrum. Consequently, an increase in flow velocity only elevates the excitation energy, thereby increasing the peak amplitudes, but does not alter the positions of these peaks, as shown in Figure 6.

In addition, we believe that the energy of turbulent pulsating pressure is primarily concentrated in the low-frequency range. Furthermore, when pressure acts on the pipe wall material, the vibration response is more pronounced in the low-frequency range because the frequencies of the pipe wall’s low-order bending modes are typically lower.

Considering the influence of wellbore diameter, the 25 mm wellbore at average flow velocities of 0.6055 m/s, 0.6564 m/s, and 0.8036 m/s corresponds to the 50 mm wellbore at average flow velocities of 0.1528 m/s, 0.1684 m/s, and 0.2009 m/s, respectively, with very similar volumetric flow rates of 1.07 m^3^/h, 1.17 m^3^/h, and 1.42 m^3^/h. From Figure 4 and Figure 6, it can be observed that the low-frequency band energy monitored in the 50 mm wellbore is slightly lower than that in the 25 mm wellbore. This may be attributed to the smaller flow cross-section in the smaller-diameter wellbore, which enhances interactions between the fluid itself and between the fluid and the pipe wall.

### 4.3. Effect of Flow Velocity on FBE

By converting DAS acoustic signals into acoustic energy and applying one-dimensional Fourier transform, the acoustic FBE within specific frequency bands of the DAS signal can be obtained. The primary issue with traditional fixed-bandwidth division methods (such as equal-width) lies in their subjective and predetermined nature. This approach sets the band boundaries prior to analysis without considering the actual distribution characteristics of the signal’s energy in the frequency domain under different flow velocities and pipe diameters. Consequently, key energy-concentrated frequency bands may be artificially fragmented, or noise-dominated bands may be mixed with characteristic bands, thereby depriving subsequent analysis of precise physical foundations.

**Figure 7:**
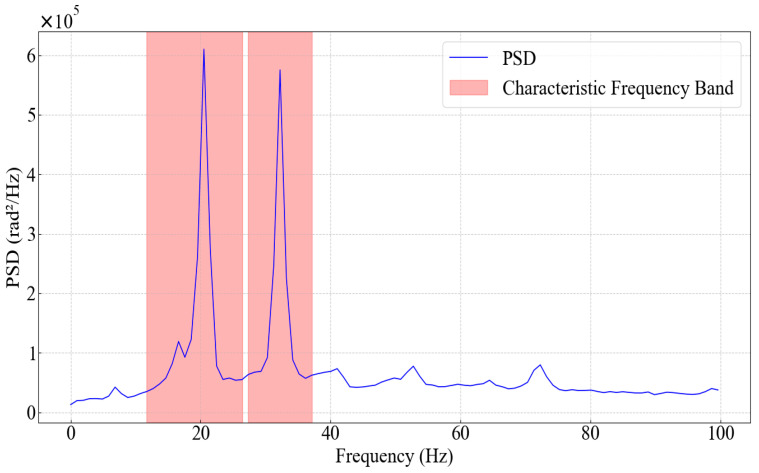
Identification of characteristic frequency bands for the 25 mm wellbore at a flow velocity of 0.8036 m/s.

Flow-induced excitation energy is not uniformly distributed in the frequency domain, but rather concentrated in certain continuous regions where variations are particularly pronounced. By identifying these regions, it is possible to objectively extract frequency bands strongly correlated with flow velocity and use them as core units for analysis, thereby laying a solid foundation for establishing reliable interpretation models. The frequency range partitioning method used in this paper is as follows: First, the adaptive partitioning of characteristic frequency bands based on energy difference statistics described in Section 3.2 is employed. Figure 7 illustrates the partitioning of characteristic frequency bands for a 25 mm borehole under a flow velocity of 0.8036 m/s (parameters α = 0.6, *β* = 0.1). After determining the characteristic frequency bands, the remaining frequency range was divided using the 1/3-octave band method, an internationally recognized standard in the fields of acoustics and vibration analysis. Widely applied in noise control and structural vibration assessment, this method sets the upper frequency limit of each band to approximately 2^1/3^ ≈ 1.26 times the lower frequency limit. Adopting this standard ensures the standardization of the research results.

**Figure 8:**
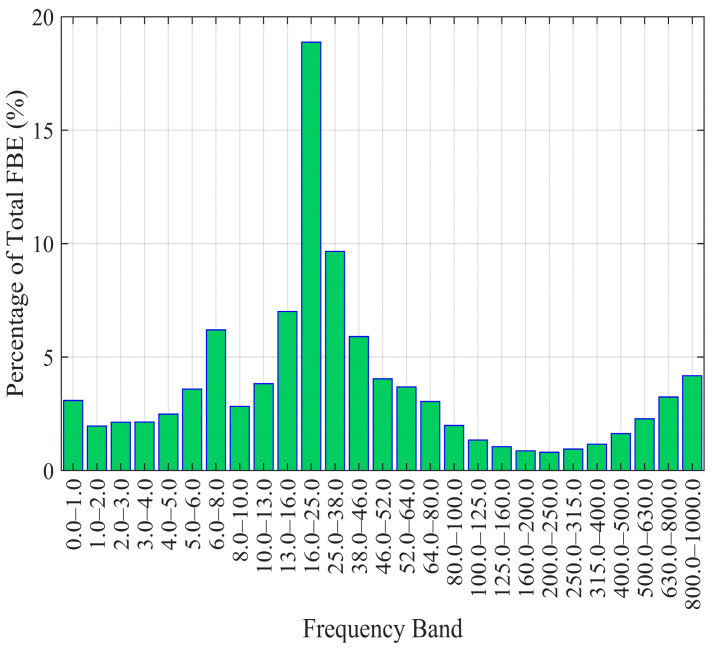
Energy proportion of each frequency band for the 25 mm wellbore at a flow velocity of 0.6055 m/s.

The primary characteristic bands for flow in the 25 mm wellbore were selected as 16–25 Hz and 25–38 Hz, resulting in a total of 27 bands. For the 50 mm wellbore, the primary characteristic bands were selected as 20–28 Hz, 28–40 Hz, and 54–64 Hz, also totaling 27 bands. Then, calculate the energy for each frequency band using the method described in Section 3.3. Figure 8 and Table 3 show the percentage of energy contributed by each frequency band relative to the total energy range (0–1000 Hz) and the FBE calculation results for a 25 mm borehole at a flow velocity of 0.6055 m/s. It can be seen that the FBE of the characteristic frequency bands dominates the FBE across the entire frequency range and is strongly correlated with flow velocity; the combined energy of the 20–28 Hz and 28–40 Hz bands accounts for 28% of the total.

### 4.4. Fitting Relationship Between Characteristic Frequency-Band Energy and Flow Velocity

From the analysis of the aforementioned influencing patterns, it is evident that the impact of different wellbore diameters and flow velocities on the acoustic response in horizontal wellbores varies in both degree and nature. The acoustic profile of a horizontal wellbore is simultaneously influenced by the interaction of multiple factors. To address this, we applied a signal processing and modeling workflow to the experimental data obtained under different wellbore diameter conditions, aiming to establish a quantitative interpretation model between the acoustic characteristic FBE and the wellbore flow velocity. For each set of experimental conditions (specific wellbore diameter and flow velocity), based on the characteristic bands selected earlier, data along the monitoring fiber was extracted and its corresponding FBE value was calculated, thereby constructing a dataset with FBE as the characteristic variable.

Processing the extensive experimental data revealed that the flow velocity and the acoustic frequency-band energy extracted from DAS data often exhibit correlations of binomial, trinomial, or exponential types. To verify the optimal mapping relationship between the characteristic-band FBE and flow velocity, regression analysis was employed to establish a mathematical relationship between FBE and the average flow velocity. By comparing the R^2^ values and parameter characteristics of seven different models, the physical rationality of the energy-velocity relationship and the applicability of each model under different frequency bands were evaluated. This analysis aims to provide a theoretical basis for flow velocity inversion based on acoustic signals.

In the 25 mm wellbore flow experiment, a focused comparison was made between the fitting performance of the characteristic frequency bands (16–25 Hz, 25–38 Hz, and their merged band 16–38 Hz), several conventional broadbands (0–100 Hz, 0–200 Hz, 0–1000 Hz), as well as the mid-frequency band (100–500 Hz) and high-frequency band (500–1000 Hz). It should be noted that the “characteristic frequency bands” used in the subsequent analysis of this paper were all identified using adaptive methods, whereas the “conventional frequency bands” (such as those in Table 2 excluding the characteristic frequency bands) were standardized based on 1/3-octave bands. The selection of several wide-band (0–100 Hz, 0–200 Hz), mid-band (100–500 Hz), high-band (500–1000 Hz), and 0–1000 Hz (covering the entire effective frequency range of DAS signals) bands for the subsequent fitting and modeling comparison analysis was intended to highlight the superiority and stability of the method used in this study.

Table 4 presents detailed statistical results of the fitting for the 25 mm wellbore. FBE represents the energy of the corresponding frequency band (The unit is rad^2^), *V* is the flow velocity (The unit is m/s), and *a* and *b* are the fitting coefficients.

**Figure 9:**
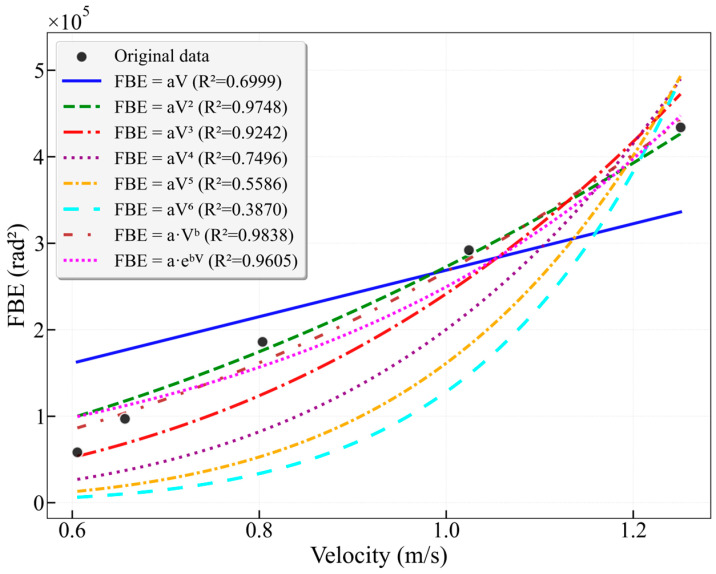
Fitting curves of FBE versus flow velocity for the characteristic frequency band 16–25 Hz in the 25 mm wellbore.

The results in Figure 9 show that among all models for the 16–25 Hz frequency band, the power-law model achieves the highest R^2^ value of 0.9838, demonstrating excellent fitting performance. The power-law exponent b = 2.2491, which is close to 2, suggests that the energy in this band may be related to the square of the flow velocity. This is followed by the quadratic model (R^2^ = 0.9748) and the exponential model (R^2^ = 0.9605). The linear model yields an R^2^ of only 0.6999, significantly lower than that of the nonlinear models, indicating that the relationship between FBE and flow velocity in this band clearly deviates from linearity. It is noteworthy that as the polynomial order increases from cubic to sextic, the R^2^ values consistently decrease (0.9242 → 0.3870), demonstrating that a higher polynomial degree does not necessarily lead to a better fit.

**Figure 10:**
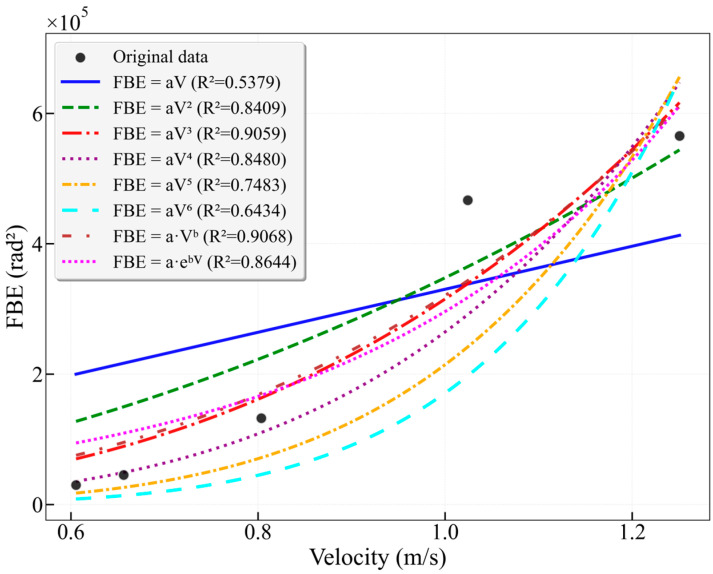
Fitting curves of FBE versus flow velocity for the characteristic frequency band 25–38 Hz in the 25 mm wellbore.

Figure 10 shows that the optimal fitting models for the 25–38 Hz frequency band are the power-law model (R^2^ = 0.9068) and the cubic model (R^2^ = 0.9059). The exponent for the power-law fit is b = 2.8823, which is higher than the 2.249 observed for the 16–25 Hz band. This indicates that the energy in this band increases with flow velocity at a faster rate, approximately related to the cube of the velocity, which explains why the cubic model demonstrates good fitting performance. The linear fit yields a very low R^2^ of 0.5379, indicating a weak linear correlation. Similarly to the 16–25 Hz band, the model fit deteriorates beyond the cubic order.

**Figure 11:**
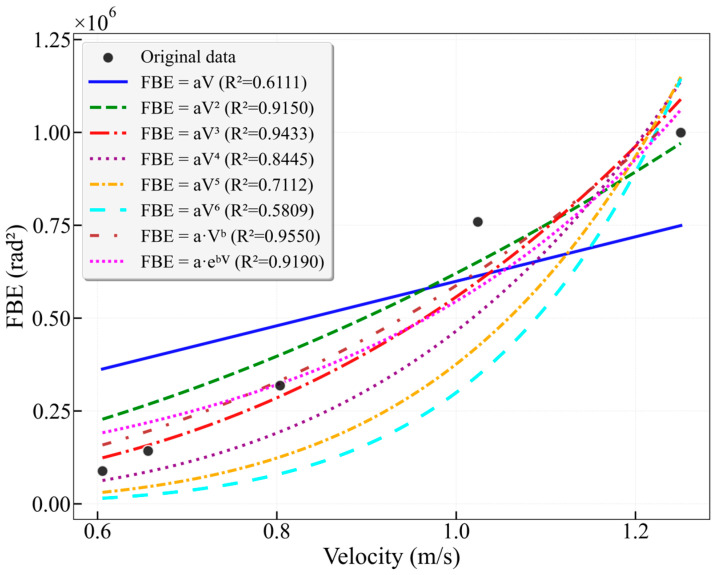
Fitting curves of FBE versus flow velocity for the characteristic frequency band 16–38 Hz in the 25 mm wellbore.

As shown in the results of Figure 11, the fitting performance for the merged 16–38 Hz frequency band lies between those of the two individual sub-bands. The optimal models are the power-law model (R^2^ = 0.9550) and the cubic model (R^2^ = 0.9433). The power-law exponent b = 2.6147 closely approximates the average of the exponents from the two sub-bands, indicating that the energy response of the merged band represents a composite of the characteristics from both individual bands. However, merging the two characteristic bands captures a more comprehensive flow signal. When using the cubic model, the goodness of fit for the merged band (R^2^ = 0.9433) is higher than that for the individual characteristic bands 16–25 Hz (R^2^ = 0.9242) and 25–38 Hz (R^2^ = 0.9059). This approach enhances the model’s robustness under varying flow conditions or minor environmental fluctuations by integrating more effective information while maintaining high accuracy.

**Figure 12:**
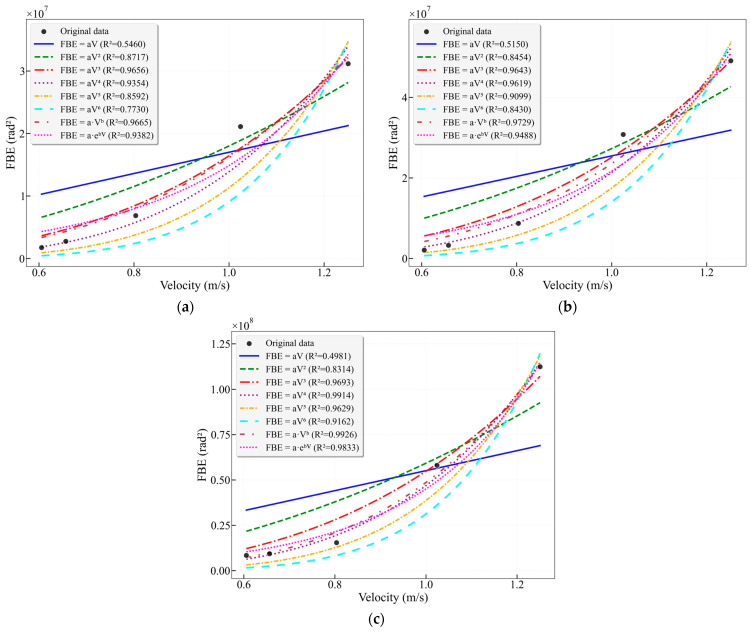
(**a**) Fitting curves of FBE versus flow velocity for the characteristic frequency band 0–100 Hz in the 25 mm wellbore; (**b**) Fitting curves of FBE versus flow velocity for the frequency band 0–200 Hz in the 25 mm wellbore; (**c**) Fitting curves of FBE versus flow velocity for the frequency band 0–1000 Hz in the 25 mm wellbore.

Traditionally, broadband analysis is employed with the intention of capturing the entire signal energy as comprehensively as possible. The 0–1000 Hz frequency band covers the entire effective frequency range of DAS signals, and its FBE represents the total energy of the flow-induced acoustic response. Figure 12 presents the fitting results of FBE versus flow velocity for the conventional broadbands (0–100 Hz, 0–200 Hz) and the full frequency band (0–1000 Hz), respectively. For the 0–100 Hz band, the best-fitting models are the power-law model (R^2^ = 0.9665) and the cubic model (R^2^ = 0.9656). For the 0–200 Hz band, the best-fitting models are the power-law model (R^2^ = 0.9729) and the cubic model (R^2^ = 0.9643). For the 0–1000 Hz full band, the best-fitting models are the power-law model (R^2^ = 0.9926) and the quartic model (R^2^ = 0.9914).

Under broadband conditions, higher-order models (cubic and above) achieve higher final R^2^ values compared to their counterparts using characteristic frequency bands. However, this apparent higher precision requires cautious evaluation. Wide bandwidth encompasses a vast array of frequency components spanning from low to high frequencies, inevitably containing a significant amount of non-flow-induced vibration signals unrelated to the core flow process, which may obscure the flow velocity-signal relationship of the core. This amalgamation can mask the fundamental flow-velocity-to-signal relationship. The exceptionally high R^2^ values achieved by higher-order models (quartic, quintic) are largely attributable to their simultaneous overfitting of these signals, rather than solely fitting the genuine flow-induced signal.

Furthermore, a notable phenomenon is the dramatic escalation in the magnitude of broadband model parameters. For instance, comparing the 16–25 Hz band (cubic model coefficient *a* = 556,758) with the 0–1000 Hz band (cubic model coefficient *a* = 54,794,449), the coefficient *a* in the latter increases by two orders of magnitude. Similarly, the power-law exponent *b* surges from 2.6147 to 3.7942. This parameter inflation does not stem from a stronger physical relevance but rather from the fact that the dependent variable, FBE, itself attains enormously large values due to the accumulation of broadband noise. Consequently, the models are forced to adjust their parameters to accommodate this vast numerical range, leading to a severe blurring of their physical interpretability.

Concurrently, the performance of low-order models is generally poor. The R^2^ values for linear models under all broadband conditions are below 0.55, and quadratic polynomials only reach 0.83–0.87. This performance is significantly inferior to that of quadratic models under characteristic frequency bands, which can achieve R^2^ values as high as 0.97.

**Figure 13:**
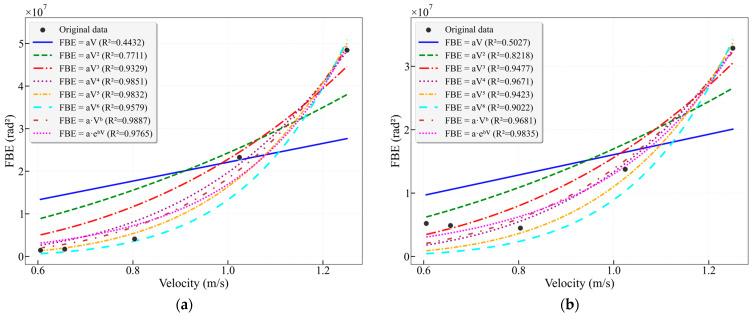
(**a**) Fitting curves of FBE versus flow velocity for the characteristic frequency band 100–500 Hz in the 25 mm wellbore; (**b**) Fitting curves of FBE versus flow velocity for the frequency band 500–1000 Hz in the 25 mm wellbore.

Further analysis of the effects in the mid-to-high frequency range selected the intermediate frequency band (100–500 Hz) and high-frequency band (500–1000 Hz), as shown in Figure 13. For the 100–500 Hz band, the power-law model achieved an R^2^ as high as 0.9887 with an exponent *b* = 4.4118, which is significantly higher than that of the low-frequency characteristic bands. The quartic model also provided a good fit with an R^2^ of 0.9851. For the 500–1000 Hz band, the exponential model performed best (R^2^ = 0.9835), while the power-law model yielded an R^2^ of 0.9681 with an exponent *b* = 3.7884. Although this exponent remains higher than those in the low-frequency range, it is slightly lower than that of the 100–500 Hz band. The quartic model followed closely with an R^2^ of 0.9671.

**Figure 14:**
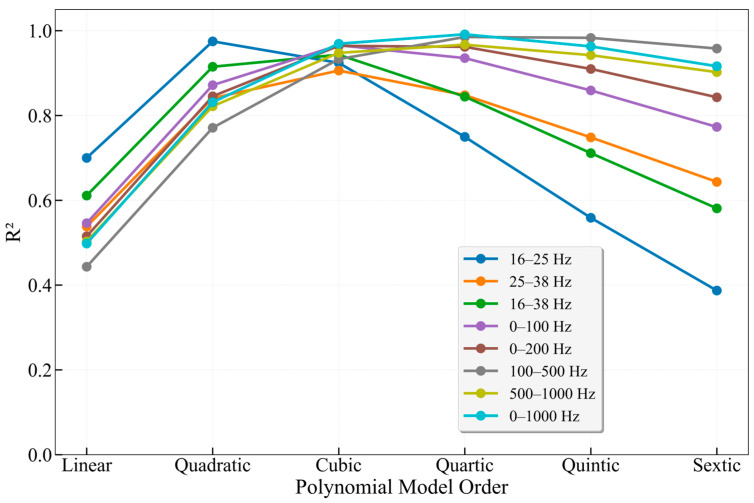
Comparison of fitting results across different frequency bands for the 25 mm wellbore.

Figure 14 presents a summary of the fitting results for different frequency bands in the 25 mm wellbore. A comprehensive comparison of the fitting results across all frequency bands reveals that the underlying relationship between FBE and flow velocity V essentially approximates a cubic power-law function, rather than a simple linear relationship. The highest R^2^ values were achieved in the fittings for the 25–38 Hz, 16–38 Hz, 0–100 Hz, and 0–200 Hz bands. For the 0–1000 Hz band, the cubic model yielded an R^2^ of 0.9693, while the quartic model achieved an R^2^ of 0.9914, a difference of only 2.28%. Furthermore, the strategy of using characteristic frequency bands for flow velocity fitting proves superior to conventional broadband methods. This advantage stems from the high signal-to-noise ratio and clear physical correlation of these characteristic bands, which are closely associated with flow-induced excitation. Notably, the 16–38 Hz band utilizes only 2.2% of the frequency range of the 0–1000 Hz band, yet its fitting results differ by merely 1.8%.

In the 50 mm wellbore flow experiment, a focused comparison was made between the fitting performance of the characteristic frequency bands (20–28 Hz, 28–40 Hz, 54–64 Hz, their merged band 20–40 Hz, and the combined band of 20–40 Hz and 54–64 Hz), several conventional broadbands (0–100 Hz, 0–200 Hz, 0–1000 Hz), as well as the mid-frequency band (100–500 Hz) and high-frequency band (500–1000 Hz). Table 5 below presents detailed statistical results of the fitting for the 50 mm wellbore. FBE represents the energy of the corresponding frequency band (The unit is rad^2^), *V* is the flow velocity (The unit is m/s), and *a* and *b* are the fitting coefficients.

**Figure 15:**
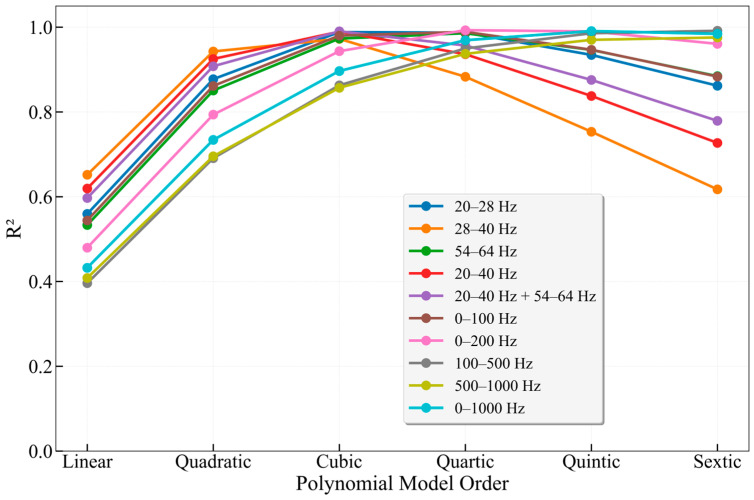
Comparison of fitting results across different frequency bands for the 50 mm wellbore.

As illustrated in Figure 15, some findings remain consistent with the previous experimental results obtained from the 25 mm wellbore. Across all frequency bands, the linear model yielded the lowest R^2^ values. The three characteristic frequency bands (20–28 Hz, 28–40 Hz, and 54–64 Hz) all exhibited a strong nonlinear relationship between FBE and flow velocity, further confirming the absence of a linear correlation between FBE and V. In contrast, nonlinear models generally demonstrated superior fitting performance. Among these, the power-law model best captured the variation in energy with flow velocity across different frequency bands; however, its exponent varies with wellbore diameter and band selection, and its physical interpretation warrants further investigation. In addition to the power-law model, the cubic model also exhibited high goodness-of-fit, performing optimally across nearly all characteristic and merged characteristic bands, while generally maintaining satisfactory fitting results in broadbands as well. Whether in the small-diameter (25 mm) or large-diameter (50 mm) wellbore, a stable cubic relationship was observed between FBE and flow velocity within the characteristic frequency bands. Moreover, by merging physically correlated characteristic bands, both the fitting accuracy and robustness of the cubic model were simultaneously enhanced.

In stark contrast to the characteristic frequency bands, the analysis results from conventional broadbands (0–100 Hz, 0–200 Hz, 0–1000 Hz) exhibited a strong diameter-dependent behavior, with the optimal fitting model varying inconsistently across different wellbores and band selections. For the 25 mm wellbore, the best-fitting model for the full 0–1000 Hz band was the quartic model (R^2^ = 0.9914), whereas for the 50 mm wellbore, the optimal model for the same band shifted to the quintic model (R^2^ = 0.9908), and the exponent of the power-law model increased sharply from 3.7942 to 5.1842. For the mid- and high-frequency bands (100–500 Hz and 500–1000 Hz), the optimal model for the 25 mm wellbore was the quartic model, while the 50 mm wellbore corresponded to the sextic model. This cross-diameter inconsistency arises from the inclusion in broadbands of substantial amounts of flow-irrelevant environmental noise and instrumental structural noise, whose distribution and intensity vary with wellbore diameter. Consequently, high-order models are forced to adjust their parameters to fit these signals, thereby obscuring the true flow velocity–energy relationship.

More notably, the optimization effect of the characteristic band merging strategy is significant. After merging the adjacent 20–28 Hz and 28–40 Hz bands into a single 20–40 Hz band, the R^2^ of the cubic model increased to 0.9895. When further incorporating the 54–64 Hz band—which likely represents distinct vibration modes in a physical sense—the cubic model achieved the highest R^2^ among all characteristic bands at 0.9903. This fitting performance even surpassed that of using the full frequency band (0–1000 Hz), which yielded an R^2^ of 0.8967. This demonstrates that selectively merging multiple physically relevant characteristic bands can more comprehensively capture the distribution characteristics of flow-induced energy. This strategy achieves a simultaneous improvement in fitting accuracy and model robustness without excessively increasing data complexity. Therefore, we recommend selecting characteristic frequency bands whenever possible in practical applications.

Comparing the results of the 25 mm and 50 mm wellbores, the differences in model coefficients primarily stem from variations in function form, energy scale, and geometric dimensions. Firstly, different models (such as linear, quadratic, power law, etc.) have varying dependence indices on flow velocity, inevitably leading to differences in fitting coefficients. Secondly, the energy scale of FBE in different frequency bands can vary by 2–3 orders of magnitude, and even with the same function form, coefficients will change with the energy scale. Furthermore, the geometric dissimilarity caused by differences in wellbore diameter (the cross-sectional area ratio of 25 mm to 50 mm pipe diameters is 1:4) is also reflected in the coefficients through structural, fluid–structure coupling efficiency, and strain transfer characteristics. Therefore, the variability of model parameters is a comprehensive representation of the aforementioned mathematical and physical factors.

### 4.5. Key Findings

In summary, the core findings are consistent across experiments with two different pipe diameters:

(1) Characteristic bands in the relatively low-frequency range (<100 Hz) consistently exhibit a strong correlation with flow-induced excitation, indicating that the dominant vibrational energy is concentrated within this frequency range. We believe that the energy of turbulent pulsating pressure is primarily concentrated in the low-frequency range. Furthermore, when pressure acts on the pipe wall material, the vibration response is more pronounced in the low-frequency range be-cause the frequencies of the pipe wall’s low-order bending modes are typically lower.
(2) In practical applications, it is best to use characteristic frequency bands. Selectively merging multiple physically related characteristic bands enables a more comprehensive capture of the distribution characteristics of flow-induced energy. This strategy achieves a simultaneous improvement in fitting accuracy and model robustness without excessively increasing data complexity. In principle, the criteria for merging should follow the principle of physical relevance—the frequency bands to be merged should originate from the same physical mechanism or similar excitation sources. In this study, the criteria for merging include: Proximity of the frequency bands in the frequency domain (adjacent or with a small frequency gap); Similarity in the shape of the frequency bands on the PSD curve (e.g., both approximately exhibiting a single-peak or double-peak structure).
(3) The relationship between FBE and flow velocity is unequivocally nonlinear, with linear models demonstrating poor fitting performance. The power-law model serves as the optimal model for characterizing the relationship between energy within each characteristic band and flow velocity. However, the physical interpretation of its exponent and its variation patterns with pipe diameter and band selection require further investigation. The cubic model (FBE ∝ *V*^3^) can serve as an effective alternative for characteristic frequency band analysis in practical applications. The discovery of this relationship hinges on abandoning the conventional approach of analyzing artificially predefined broadband or full-band energy. Instead, the focus shifts to characteristic frequency components that are physically excited by the flow. This fundamental shift results in an intrinsic enhancement of the signal-to-noise ratio and enables the development of highly accurate and robust flow velocity models.

## 5. Discussion

### 5.1. Research Comparison

Our findings stand in striking contrast to the acoustic patterns reported in perforation scenarios, as shown in Table 6. Existing research indicates that when fluid flows through perforation holes, the generated sound pressure level (SPL) exhibits a linear relationship with flow rate, expressed as SPL ∝ log(*q*^3^), and the SPL can be represented by the corresponding FBE [21].

These two relationships are not contradictory but rather profoundly reflect the underlying physics under different flow geometries and excitation mechanisms. The DAS signals investigated in this study, originating from flow-induced pipe-wall vibration in pipe/wellbore internal flows, exhibit a power-law relationship between characteristic band energy and flow velocity. This reflects the nonlinear process by which turbulent fluctuation energy couples into mechanical vibrations through specific structural modes. In contrast, perforation studies measure the broadband acoustic waves directly radiated by turbulent jets, the characteristics of which follow acoustic scaling laws manifesting on a logarithmic scale.

### 5.2. Mechanism Explanation

Previous studies have shown that fluid flow noise represents the pulsating kinetic energy within a turbulent energy field, which is converted into acoustic energy and radiated outward through fluid–structure/fluid-acoustic coupling. The radiated noise generated by fluid flow is a result of the turbulent flow field [42]. The higher the rate at which mechanical energy is dissipated into turbulence, the greater the radiated noise power, that is:

(20) $$P_{n}=F\left(\frac{d\varepsilon }{dt}\right)$$

where *P_n_* represents the noise power amplitude and *ε* represents the mechanical energy dissipated into turbulence.

We assume that the total energy dissipation rate reflects the turbulent energy field, therefore:

(21) $$\frac{d\varepsilon }{dt}\sim \Delta p\cdot q$$

where Δ*p* is the pressure drop and *q* is the volumetric flow rate.

Combining the relationship between the Δ*p* and the flow velocity *u*:

(22) $$\Delta p=\lambda \cdot \frac{L}{D}\cdot \frac{\rho u^{2}}{2}$$

where *λ* is the friction coefficient, *L* is the pipe length, *D* is the pipe diameter, and *ρ* is the fluid density.

This study uses single-phase water as the experimental medium. Under our experimental conditions, the Reynolds number (Re = *ρuD*/*μ*, *μ* is the viscosity of water) ranges from 7000 to 32,000. Within this range, the flow is in a turbulent state, and the inner wall of the pipe can be considered a hydraulically smooth surface. The relationship between *λ* and Re is as follows [43]:

(23) λ=0.3164Re0.25

Therefore, there is

(24) Δp∝0.3164Re0.25⋅u2∝u1.75 

The final result is

(25) $$P_{n}\propto \Delta p\cdot q\propto u^{1.75}\cdot u=u^{2.75}$$

The signals measured by DAS are essentially responses to sound pressure fluctuations in a fluid noise field, while noise power represents the total acoustic energy output of the noise field; this conclusion is consistent with the findings of this study.

## 6. Conclusions

This study conducted experimental research based on a horizontal wellbore fluid flow fiber-optic monitoring simulation system to systematically investigate the influence of flow velocity on DAS signals under pure water injection conditions in different pipes. The proposed workflow of “PSD Analysis—Characteristic Band Delineation—FBE Fitting” effectively extracts flow-velocity-sensitive acoustic features from DAS signals and establishes stable and reliable quantitative interpretation models. By delineating characteristic frequency bands based on power spectral density, this approach replaces traditional subjective band partitioning. Subsequently, characteristic band energy was extracted, and the fitting performance of eight mathematical models was systematically compared to identify the optimal relationship model between flow velocity and DAS acoustic FBE. The main conclusions of this study are as follows:

(1) The frequency-domain distribution characteristics under different wellbore diameters and flow velocities exhibit significant differences. For the small-diameter wellbore (25 mm), characteristic frequencies are primarily concentrated in the low-frequency region below 50 Hz, with a relatively narrow band and a smooth energy distribution curve, showing a dominant dual-peak feature. For the large-diameter wellbore (50 mm), while maintaining low-frequency dominance, the energy begins to extend into the 50–200 Hz band, and multiple characteristic frequency peaks appear. The low-frequency band (<100 Hz) is strongly correlated with flow and dominates the energy distribution. As flow velocity increases, the acoustic energy shows an increasing trend, and the energy concentration band gradually broadens.
(2) Adaptive methods based on energy difference statistics for identifying and delineating feature bands can extract frequency bands associated with different flow intensities. In practical applications, characteristic frequency bands should be used whenever possible, as they overcome the limitations of traditional fixed-band segmentation, which fails to account for the actual frequency distribution characteristics of specific signals. Traditional methods often result in artificial fragmentation of true feature bands or the conflation of noise bands with feature bands.
(3) The relationship between the FBE of DAS signals and flow velocity is significantly nonlinear. The power-law model serves as the theoretically optimal fitting model. However, its exponent varies with experimental setup, frequency band, etc., and its physical interpretation requires further in-depth study. The cubic model (FBE ∝ *V*^3^) can serve as an effective alternative for characteristic frequency band analysis in practical applications. Furthermore, employing a characteristic band merging strategy allows for a more comprehensive capture of the distribution characteristics of flow-induced energy, achieving simultaneous improvement in fitting accuracy and model robustness without excessively increasing data complexity. Finally, the relationship between energy and flow velocity derived using fluid dynamics is in good agreement with the results of this study.
(4) Future research could extend to multiphase flow, different wellbore configurations, and wider flow-velocity ranges to further validate the universality and robustness of the model. More rigorous control and real-time monitoring of water temperature, together with quantitative degassing or bubble-monitoring procedures, would help decouple the effects of fluid thermophysical properties and entrained gas on DAS acoustic propagation. In addition, CFD simulations could be employed to reveal the intrinsic vibration modes and flow-field structures under specific operating conditions, thereby establishing a more complete physical connection between microscopic flow behavior and macroscopic DAS signal characteristics. For field-scale applications, further studies should also consider the influence of longer well lengths, heterogeneous formation conditions, imperfect fiber coupling, and external operational noise and combine site-specific calibration with normalized band-energy analysis to improve the transferability of the laboratory-derived interpretation framework.

## Figures and Tables

**Table 1:** Experimental test matrix.

Wellbore Inner Diameter (mm)	Pump Speed (rpm)	Flow Rate (m^3^/h)	Average Flow Velocity (m/s)
25	1100	1.06~1.08	0.6055
	1200	1.16~1.17	0.6564
	1400	1.37~1.47	0.8036
	1800	1.79~1.84	1.0242
	2200	2.20~2.22	1.2506
50	1100	1.08~1.09	0.1528
	1200	1.19~1.20	0.1684
	1400	1.42~1.43	0.2009
	1600	1.64~1.65	0.2320
	1800	1.86~1.87	0.2631
	2000	2.09~2.10	0.2957
	2200	2.34~2.36	0.3325
	2400	2.57~2.58	0.3636

**Table 2:** Comparison of Other Methods with FBE.

Feature Type	Limitations	Advantages of FBE
Sound pressure level (SPL)	① It can only reflect the overall sound pressure level; ② It is susceptible to environmental noise and measurement gain, resulting in significant fluctuations in amplitude; ③ It cannot capture the frequency-band energy distribution characteristics corresponding to different flow structures within the flow field.	① FBE can accurately quantify the energy distribution across specific frequency bands and can eliminate the effects of measurement gain and environmental noise through preprocessing; ② It directly reflects the energy distribution patterns of different flow structures and is highly coupled with the underlying physical mechanisms.
Time-frequency characteristics	① High-dimensional data and high computational costs; ② The physical significance of the time-frequency coefficients is ambiguous, making it difficult to directly link them to the physical mechanisms of flow structures; ③ Susceptible to the effects of time window and wavelet basis selection, resulting in poor feature stability.	① FBE is a low-dimensional feature with a clear physical meaning; it is computationally efficient and suitable for online monitoring; ② The energy in each frequency band directly corresponds to the vibration or acoustic emission energy of the flow structure, making it interpretable; ③ Feature extraction relies solely on PSD integration.
Wavelet energy	① Wavelet energy depends on the choice of wavelet basis and the number of decomposition levels; ② The division of wavelet frequency bands has no clear physical meaning; ③ The computational complexity is high, which is not conducive to online engineering applications.	① The frequency band division of FBE is based on the characteristic flow frequency, which has a clear physical meaning; ② The calculation process is simple, making it suitable for online monitoring at engineering sites and thus more practical.

**Table 3:** Calculated FBE results for the 25 mm wellbore at a flow velocity of 0.6055 m/s.

Band Index	Band Range (Hz)	FBE (rad^2^)
1	0.0–1.0	9525.50
2	1.0–2.0	6062.05
3	2.0–3.0	6578.70
4	3.0–4.0	6595.83
5	4.0–5.0	7673.95
6	5.0–6.0	11,069.38
7	6.0–8.0	19,119.71
8	8.0–10.0	8733.71
9	10.0–13.0	11,815.51
10	13.0–16.0	21,619.81
11	16.0–25.0	58,250.21
12	25.0–38.0	29,801.12
13	38.0–46.0	18,234.31
14	46.0–52.0	12,480.74
15	52.0–64.0	11,379.21
16	64.0–80.0	9395.16
17	80.0–100.0	6135.56
18	100.0–125.0	4142.63
19	125.0–160.0	3246.13
20	160.0–200.0	2687.82
21	200.0–250.0	2473.09
22	250.0–315.0	2936.17
23	315.0–400.0	3594.10
24	400.0–500.0	5040.71
25	500.0–630.0	7041.00
26	630.0–800.0	10,005.98
27	800.0–1000.0	12,876.80

**Table 4:** Fitting results of FBE and flow velocity for different frequency bands in the 25 mm wellbore.

Feature Frequency Band	Formula	Parameters	R^2^
16–25 Hz	FBE *= aV*	*a* = 268,726	0.6999
	FBE *= aV*^2^	*a* = 272,690	0.9748
	FBE *= aV*^3^	*a* = 241,546	0.9242
	FBE *= aV*^4^	*a* = 200,243	0.7496
	FBE *= aV*^5^	*a* = 161,230	0.5586
	FBE *= aV*^6^	*a =* 128,199	0.3870
	FBE *= a·V^b^*	*a* = 267,079, *b* = 2.2491	0.9838
	FBE *= a·e^bV^*	*a* = 24,290, *b* = 2.3295	0.9605
25–38 Hz	FBE *= aV*	*a* = 330,153	0.5379
	FBE *= aV*^2^	*a* = 347,781	0.8409
	FBE *= aV*^3^	*a* = 315,212	0.9059
	FBE *= aV*^4^	*a* = 264,685	0.8480
	FBE *= aV*^5^	*a* = 214,442	0.7483
	FBE *= aV*^6^	*a =* 170,868	0.6434
	FBE *= a·V^b^*	*a* = 320,501, *b* = 2.8823	0.9068
	FBE *= a·e^bV^*	*a* = 16,324, *b* = 2.8976	0.8644
16–38 Hz	FBE *= aV*	*a* = 598,879	0.6111
	FBE *= aV*^2^	*a* = 620,471	0.9150
	FBE *= aV*^3^	*a* = 556,758	0.9433
	FBE *= aV*^4^	*a* = 464,928	0.8445
	FBE *= aV*^5^	*a* = 375,672	0.7112
	FBE *= a·V*^6^	*a* = 299,066	0.5809
	FBE *= a·V^b^*	*a* = 587,303, *b* = 2.6147	0.9550
	FBE *= a·e^bV^*	*a* = 38,229, *b* = 2.6571	0.9190
0–100 Hz	FBE *= aV*	*a* = 17,002,110	0.5460
	FBE *= aV*^2^	*a* = 18,007,267	0.8717
	FBE *= aV*^3^	*a* = 16,427,471	0.9656
	FBE *= aV*^4^	*a* = 13,888,407	0.9354
	FBE *= aV*^5^	*a* = 11,326,683	0.8592
	FBE *= a·V*^6^	*a =* 9,080,650	0.7730
	FBE *= a·V^b^*	*a* = 16,129,391, *b* = 3.1275	0.9665
	FBE *= a·e^bV^*	*a* = 646,203, *b* = 3.1367	0.9382
0–200 Hz	FBE *= aV*	*a* = 25,468,410	0.5150
	FBE *= aV*^2^	*a* = 27,279,055	0.8454
	FBE *= aV*^3^	*a* = 25,097,401	0.9643
	FBE *= aV*^4^	*a* = 21,354,576	0.9619
	FBE *= aV*^5^	*a* = 17,501,137	0.9099
	FBE *= a·V*^6^	*a* = 14,083,986	0.8430
	FBE *= a·V^b^*	*a* = 23,555,646, *b* = 3.4331	0.9729
	FBE *= a·e^bV^*	*a* = 712,201, *b* = 3.4163	0.9488
100–500 Hz	FBE *= aV*	*a* = 22,110,590	0.4432
	FBE *= aV*^2^	*a* = 24,292,761	0.7711
	FBE *= aV*^3^	*a* = 22,802,924	0.9329
	FBE *= aV*^4^	*a* = 19,712,098	0.9851
	FBE *= aV*^5^	*a* = 16,359,434	0.9832
	FBE *= a·V*^6^	*a* = 13,298,092	0.9579
	FBE *= a·V^b^*	*a* = 18,318,768, *b* = 4.4118	0.9887
	FBE *= a·e^bV^*	*a* = 236,867, *b* = 4.2697	0.9765
500–1000 Hz	FBE *= aV*	*a* = 16,054,286	0.5027
	FBE *= aV*^2^	*a* = 16,974,635	0.8218
	FBE *= aV*^3^	*a* = 15,598,769	0.9477
	FBE *= aV*^4^	*a* = 13,338,106	0.9671
	FBE *= aV*^5^	*a* = 11,014,081	0.9423
	FBE *= a·V*^6^	*a* = 8,936,775	0.9022
	FBE *= a·V^b^*	*a* = 13,839,345, b = 3.7884	0.9681
	FBE *= a·e^bV^*	*a* = 333,101, b = 3.6657	0.9835
0–1000 Hz	FBE *= aV*	*a* = 55,132,822	0.4981
	FBE *= aV*^2^	*a* = 59,237,535	0.8314
	FBE *= aV*^3^	*a* = 54,794,449	0.9693
	FBE *= aV*^4^	*a* = 46,908,605	0.9914
	FBE *= aV*^5^	*a* = 38,675,253	0.9629
	FBE *= a·V*^6^	*a* = 31,295,191	0.9162
	FBE *= a·V^b^*	*a* = 48,625,705, *b* = 3.7942	0.9926
	FBE *= a·e^bV^*	*a* = 1,091,600, *b* = 3.7201	0.9833

**Table 5:** Fitting results of FBE and flow velocity for different frequency bands in the 50 mm wellbore.

Feature Frequency Band	Formula	Parameters	R^2^
20–28 Hz	FBE *= aV*	*a* = 860,581	0.5594
	FBE *= aV*^2^	*a* = 3,137,298	0.8774
	FBE *= aV*^3^	*a* = 10,046,922	0.9884
	FBE *= aV*^4^	*a* = 30,085,370	0.9870
	FBE *= aV*^5^	*a* = 86,919,801	0.9346
	FBE *= aV*^6^	*a* = 246,037,922	0.8619
	FBE *= a·V^b^*	*a* = 16,527,078, *b* = 3.4484	0.9971
	FBE *= a·e^bV^*	*a* = 7018, *b* = 11.8250	0.9851
28–40 Hz	FBE *= aV*	*a* = 1,573,106	0.6518
	FBE *= aV*^2^	*a* = 5,594,256	0.9428
	FBE *= aV*^3^	*a* = 17,567,623	0.9724
	FBE *= aV*^4^	*a* = 51,800,029	0.8830
	FBE *= aV*^5^	*a* = 147,838,851	0.7533
	FBE *= aV*^6^	*a* = 414,425,991	0.6173
	FBE *= a·V^b^*	*a* = 11,749,112, *b* = 2.6400	0.9816
	FBE *= a·e^bV^*	*a* = 27,481, *b* = 9.3907	0.9584
54–64 Hz	FBE *= aV*	*a* = 810,779	0.5332
	FBE *= aV*^2^	*a* = 2,977,538	0.8505
	FBE *= aV*^3^	*a* = 9,581,572	0.9731
	FBE *= aV*^4^	*a* = 28,785,691	0.9858
	FBE *= aV*^5^	*a* = 83,351,989	0.9463
	FBE *= aV*^6^	*a* = 236,308,266	0.8845
	FBE *= a·V^b^*	*a* = 19,647,279, *b* = 3.6479	0.9895
	FBE *= a·e^bV^*	*a* = 5492, *b* = 12.4151	0.9757
20–40 Hz	FBE *= aV*	*a* = 2,433,687	0.6195
	FBE *= aV*^2^	*a* = 8,731,553	0.9251
	FBE *= aV*^3^	*a* = 27,614,545	0.9895
	FBE *= aV*^4^	*a* = 81,885,399	0.9363
	FBE *= aV*^5^	*a* = 234,758,653	0.8378
	FBE *= aV*^6^	*a* = 660,463,912	0.7270
	FBE *= a·V^b^*	*a* = 24,873,094, *b* = 2.9062	0.9900
	FBE *= a·e^bV^*	*a* = 32,910, *b* = 10.2155	0.9720
20–40 Hz + 54–64 Hz	FBE *= aV*	*a* = 3,244,466	0.5968
	FBE *= aV*^2^	*a* = 11,709,091	0.9078
	FBE *= aV*^3^	*a* = 37,196,117	0.9903
	FBE *= aV*^4^	*a* = 110,671,090	0.9568
	FBE *= aV*^5^	*a* = 318,110,642	0.8756
	FBE *= aV*^6^	*a* = 896,772,179	0.7790
	FBE *= a·V^b^*	*a* = 40,833,131, *b* = 3.0838	0.9907
	FBE *= a·e^bV^*	*a* = 37,092, *b* = 10.7533	0.9743
0–100 Hz	FBE *= aV*	*a* = 66,335,663	0.5441
	FBE *= aV*^2^	*a* = 242,672,358	0.8614
	FBE *= aV*^3^	*a* = 779,289,558	0.9801
	FBE *= aV*^4^	*a* = 2,338,950,796	0.9890
	FBE *= aV*^5^	*a* = 6,770,795,149	0.9468
	FBE *= aV*^6^	*a* = 19,198,342,779	0.8834
	FBE *= a·V^b^*	*a* = 1,503,094,049, *b* = 3.5925	0.9940
	FBE *= a·e^bV^*	*a* = 470,882, *b* = 12.2600	0.9844
0–200 Hz	FBE *= aV*	*a* = 121,582,337	0.4797
	FBE *= aV*^2^	*a* = 452,398,718	0.7937
	FBE *= aV*^3^	*a* = 1,472,639,586	0.9434
	FBE *= aV*^4^	*a* = 4,468,382,112	0.9930
	FBE *= aV*^5^	*a* = 13,049,398,525	0.9899
	FBE *= aV*^6^	*a* = 37,267,093,348	0.9607
	FBE *= a·V^b^*	*a* = 6,819,513,076, *b* = 4.3913	0.9962
	FBE *= a·e^bV^*	*a* = 420,525, *b* = 14.4941	0.9918
100–500 Hz	FBE *= aV*	*a* = 137,116,105	0.3960
	FBE *= aV*^2^	*a* = 522,285,346	0.6909
	FBE *= aV*^3^	*a* = 1,731,981,326	0.8629
	FBE *= aV*^4^	*a* = 5,333,247,106	0.9498
	FBE *= aV*^5^	*a* = 15,758,831,490	0.9856
	FBE *= aV*^6^	*a* = 45,429,237,599	0.9915
	FBE *= a·V^b^*	*a* = 35,387,314,115, b = 5.7625	0.9921
	FBE *= a·e^bV^*	*a* = 142,379, b = 18.1622	0.9881
500–1000 Hz	FBE *= aV*	*a* = 85,297,474	0.4082
	FBE *= aV*^2^	*a* = 318,953,551	0.6955
	FBE *= aV*^3^	*a* = 1,047,708,831	0.8570
	FBE *= aV*^4^	*a* = 3,211,789,947	0.9371
	FBE *= aV*^5^	*a* = 9,473,906,289	0.9701
	FBE *= aV*^6^	*a* = 27,304,166,610	0.9757
	FBE *= a·V^b^*	*a* = 21,359,778,032, b = 5.7666	0.9762
	FBE *= a·e^bV^*	*a* = 98,459, b = 17.7690	0.9825
0–1000 Hz	FBE *= aV*	*a* = 287,926,535	0.4323
	FBE *= aV*^2^	*a* = 1,080,668,040	0.7341
	FBE *= aV*^3^	*a* = 3,547,934,424	0.8967
	FBE *= aV*^4^	*a* = 10,849,266,110	0.9692
	FBE *= aV*^5^	*a* = 31,899,276,152	0.9908
	FBE *= aV*^6^	*a* = 91,627,427,485	0.9843
	FBE *= a·V^b^*	*a* = 38,798,742,108, *b* = 5.1842	0.9913
	FBE *= a·e^bV^*	*a* = 511,285, *b* = 16.5153	0.9913

**Table 6:** Comparison of Perforation-Related Literature with This Study.

Comparison Dimension	Chen et al. (2015) [21]	This Study
Research Object	Fluid flowing from fractures into the wellbore through perforation holes	Internal flow in horizontal wellbore pipelines
Experimental Setup	Fracture unit (0.2-inch width) connected to a 5.5-inch wellbore via perforation tunnels (0.364–0.622-inch diameter)	Horizontal PVC pipes (25 mm and 50 mm inner diameter) with optical fibers continuously deployed along the pipe axis
Monitoring Method	Hydrophone and microphone arrays, point measurement	Distributed Acoustic Sensing (DAS), distributed measurement
Flow Velocity	15 m/s	1 m/s
Energy-Velocity Relationship	SPL ∝ log(*q*^3^)	FBE ∝ *V^3^*

## Data Availability

The data presented in this study are not publicly available due to privacy and ethical restrictions related to participant confidentiality. Data may be made available upon request from the corresponding author.
